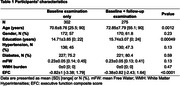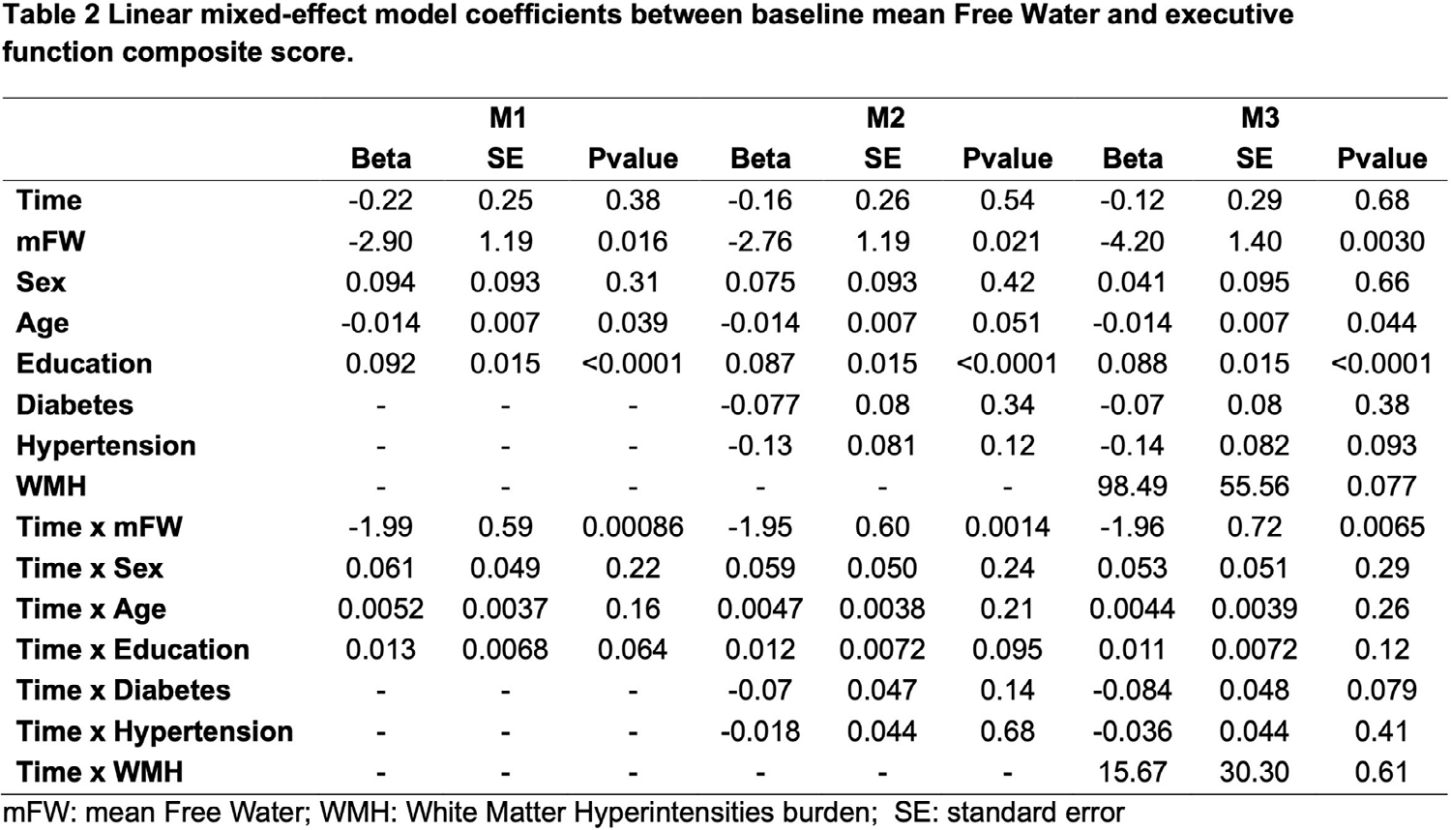# White matter free water: a sensitive biomarker of small vascular disease for VCID research

**DOI:** 10.1002/alz.086593

**Published:** 2025-01-09

**Authors:** Pauline Maillard, Hanzhang Lu, Konstantinos Arfanakis, Brian T Gold, Joel H. Kramer, Danny JJ Wang, Sudha Seshadri, Claudia L Satizabal, Gary A. Rosenberg, Herpreet Singh, Kristin Schwab, Karl HELMER, Steven M. Greenberg, Charles Decarli, Caprihan Arvind

**Affiliations:** ^1^ Alzheimer's Disease Research Center, University of California Davis, Sacramento, CA USA; ^2^ Johns Hopkins University School of Medicine, Baltimore, MD USA; ^3^ Department of Diagnostic Radiology and Nuclear Medicine, Rush UniversityRush University Medical Center, Chicago, IL USA; ^4^ University of Kentucky, Lexington, KY USA; ^5^ Memory and Aging Center, UCSF Weill Institute for Neurosciences, University of California, San Francisco, San Francisco, CA USA; ^6^ University of Southern California, Los Angeles, CA USA; ^7^ Glenn Biggs Institute for Alzheimer’s & Neurodegenerative Diseases, University of Texas Health Science Center, San Antonio, TX USA; ^8^ Glenn Biggs Institute for Alzheimer’s & Neurodegenerative Diseases, University of Texas Health Science Center at San Antonio, San Antonio, TX USA; ^9^ University of New Mexico, Albuquerque, NM USA; ^10^ Massachusetts General Hospital, Boston, MA USA; ^11^ Department of Radiology, Harvard University, Cambridge, MA USA; ^12^ University of California, Davis, CA USA; ^13^ The Mind Research Network, Albuquerque, NM USA

## Abstract

**Background:**

In recent efforts to improve early identification, staging, and prediction of risk of persons at risk for vascular contributions to cognitive impairment and dementia (VCID) in relation with small vessel disease (SVD), the MarkVCID consortium has worked to identify and validate fluid‐ and imaging‐based biomarkers for SVD associated with VCID. Free water (FW) measured derived from diffusion tensor imaging and one of the selected neuroimaging biomarker “kits”, has been demonstrated to have excellent instrumental validity and to be a sensitive biomarker of cognitive performances. We sought to further examine FW clinical relevance by investigating whether FW predicts cognitive worsening over time.

**Method:**

This study included 275 individuals from the MarkVCID‐1 cohort who received baseline MRI and clinical assessment, as well as a follow‐up clinical examination (mean time±SD: 1.31±0.49 years, Table 1). Our goal was to evaluate whether baseline mean FW (mFW) predicts change UDS3‐EF, a validated executive function composite score derived from the Uniform Data Set (v3.0). We used a linear mixed‐effect regression with EFC score as the outcome variable and time, baseline mFW, age, sex and education, as well as interactions between time and other variables as fixed effects, along with a random subject‐specific intercept and time slope (M1). In secondary models, we also adjusted for presence of diabetes, hypertension (M2) and white matter hyperintensities (log‐transformed, normalized for intracranial volume, M3) to estimate the added contribution of mFW above vascular risk factors and to and a classic SVD marker.

**Result:**

We found a significant effect of time and mFW on EFC scores (b=‐0.63, p=0.0092 and b=‐0.22, p<0.0001 respectively, Table 2), indicating that IRT scores decrease with time and increased baseline mFW. The significant interaction between time and mFW (b=‐0.079, p=0.00075), indicated that increased baseline mFW is associated with accelerated EFC decline. Adding VRF and WMH burden to model M1 did not change these relationships (Table 2).

**Conclusion:**

Our study revealed that baseline FW can predict cognitive worsening over a period as short as of 18 months, providing additional evidence of the clinical relevance of FW as a biomarker of SVD in the context of VCID research.